# Revolutionizing Inflammatory Bowel Disease Management: A Comprehensive Narrative Review of Innovative Dietary Strategies and Future Directions

**DOI:** 10.7759/cureus.44304

**Published:** 2023-08-29

**Authors:** Shahzeb Saeed, Chukwuyem Ekhator, Ali M Abdelaziz, Husnain Naveed, Amanda Karski, Daniel E Cook, Shivani M Reddy, Maryam Affaf, Salman J Khan, Sophia B Bellegarde, Abdur Rehman, Abdul Haseeb Hasan, Abdullah Shehryar

**Affiliations:** 1 Internal Medicine, Army Medical College, Rawalpindi, PAK; 2 Neuro-Oncology, New York Institute of Technology, College of Osteopathic Medicine, Old Westbury, USA; 3 Internal Medicine, Alexandria University Faculty of Medicine, Alexandria, EGY; 4 Internal Medicine, Shifa Tameer-E-Millat University Shifa College of Medicine, Islamabad, PAK; 5 Emergency Medicine, American University of Antigua, Miami, USA; 6 Medicine, Avalon University School of Medicine, Youngstown, USA; 7 Medicine, Chalmeda Anand Rao Institute of Medical Sciences, Karimnagar, IND; 8 Internal Medicine, Women’s Medical and Dental College, Abbotabad, PAK; 9 Hematology & Oncology, Mayo Clinic, Jacksonville, Florida, USA; 10 Pathology and Laboratory Medicine, American University of Antigua, St. John's, ATG; 11 Surgery, Mayo Hospital, Lahore, PAK; 12 Internal Medicine, Mayo Hospital, Lahore, PAK; 13 Internal Medicine, Allama Iqbal Medical College, Lahore, PAK

**Keywords:** nutritional interventions, ibd prevalence, disease management, dietary interventions, inflammatory bowel disease (ibd)

## Abstract

This comprehensive narrative review delves into the intricate interplay between diet and inflammatory bowel disease (IBD), shedding light on the potential impact of dietary interventions in disease management. By analyzing nutritional interventions, risks, challenges, and future perspectives, this review serves as a vital resource for clinicians, researchers, and patients alike. The amalgamation of evidence underscores the significance of customizing dietary strategies for individual patients, considering disease phenotype and cultural factors. Through an exploration of dietary components' effects on IBD, including exclusive enteral nutrition and omega-3 fatty acids, this review offers pragmatic implementation advice and outlines avenues for further research. Bridging the gap between research findings and clinical applications, the review facilitates informed decision-making and patient-centric care. In the face of escalating IBD prevalence, this review emerges as an indispensable guide for healthcare professionals, empowering them to navigate the complexities of dietary management while enabling patients to actively participate in their care trajectory. Ultimately, this narrative review advances the understanding of diet's pivotal role in IBD management, fostering a more integrated approach to patient care and paving the way for improved research and policy initiatives in the field of inflammatory bowel diseases.

## Introduction and background

Inflammatory bowel disease (IBD), encompassing Crohn's disease and ulcerative colitis, presents complex challenges that extend beyond inflammation and immune dysregulation [1,2]. Dietary interventions have gained prominence as adjunctive strategies in IBD management, offering a holistic approach to complement conventional therapies [3]. This narrative review delves into the intricate landscape of nutritional strategies in IBD management, aiming to provide insights into the role of diet in disease modulation, the mechanisms underlying dietary interventions, and the challenges and considerations that shape their implementation [4]. By examining the potential benefits, risks, and evolving research landscape, this review aims to equip healthcare professionals with a comprehensive understanding of the practical implications of dietary interventions and their integration into the multi-disciplinary management of IBD [5]. As the field of IBD management continues to evolve, elucidating the interplay between nutrition, gut health, and disease outcomes remains a crucial avenue for enhancing patient care and quality of life [6].

## Review

Systematic search and selection strategy

In our pursuit to identify relevant articles for our review, a systematic and meticulous approach was employed. We began by carrying out an extensive search within major electronic databases such as PubMed, Scopus, Web of Science, and Google Scholar, each chosen for their respective range and depth in covering scientific literature.

The foundation of our search strategy rested upon a combination of specific keywords and terms. Under the umbrella of conditions and diseases, we considered terms such as "Inflammatory Bowel Disease," "IBD," "Crohn’s Disease," "Ulcerative Colitis," "gastrointestinal disorders," and "irritable bowel syndrome (IBS)." For dietary interventions and nutrients, we focused on keywords such as "Diet," "Dietary Intervention," "Exclusive Enteral Nutrition (EEN)," "Low FODMAP," "fermentable carbohydrates," "micronutrient deficiencies," and "dietary supplements." In the realm of microbiota and metabolism, we sought articles related to "gut microbiota," "microbial diversity," "metabolome," and "short-chain fatty acids (SCFAs)." Finally, under symptoms & quality-of-life metrics, we included terms such as "functional gastrointestinal symptoms," "PedsQL," and "quality of life."

To further refine our search and zero in on the most relevant articles, we made use of Boolean operators ("AND", "OR", "NOT"). An illustrative search string could look something like this: ("Inflammatory Bowel Disease" OR "IBD" OR "Crohn’s Disease" OR "Ulcerative Colitis") AND ("Diet" OR "Dietary Intervention" OR "Low FODMAP") AND "gut microbiota." Recognizing that each database possesses its unique search functionalities, we tailored our search terms for each, ensuring we leveraged the full potential of the database in question.

Once our search yielded a collection of potential articles, our first line of assessment involved screening the titles, abstracts, and keywords. Following this initial appraisal, we engaged in a thorough evaluation of the full-text articles to decide their relevance and appropriateness for inclusion. We ensured adherence to our inclusion and exclusion criteria, with articles qualifying if they were peer-reviewed, written in English, and directly related to our research focus. Emphasis was also placed on articles from the past five years for contemporary relevance. On the contrary, articles without empirical evidence or those diverging from our core research topic were set aside. Non-peer-reviewed articles, commentaries, and editorials were similarly excluded to preserve the scientific integrity of our review. Through this rigorous process, we curated a selection of articles that provided credible, recent, and pertinent insights into our research question.

Diagnostics

Diagnostic Criteria

Inflammatory bowel disease (IBD), encompassing conditions such as Crohn's disease (CD) and ulcerative colitis (UC) [7], presents with symptoms such as abdominal pain and diarrhea [1]. The diagnosis relies on colonoscopy findings, with UC showing continuous inflammation and CD displaying patchy areas [8]. Histological evidence from biopsies, radiological studies, and laboratory tests, including blood and stool markers, aids in the diagnostic process [2]. Advanced imaging techniques, such as CT and MRI, highlight complications such as abscesses [1]. Serological markers, such as pANCA in UC and ASCA in CD, provide additional insights [3], while ruling out other causes, such as infections and colorectal cancer, is crucial [9].

Differential Diagnosis

Differential diagnoses for IBD are crucial to distinguishing it from other conditions with similar gastrointestinal symptoms. Infectious colitis, often caused by pathogens such as Clostridium difficile, can mimic IBD symptoms but typically has a more acute onset [10]. Ischemic colitis, resulting from reduced blood flow to the colon, presents with abdominal pain and bloody diarrhea, similar to IBD [11]. Diverticulitis, inflammation of the pouches in the colon, can also resemble IBD, especially in older adults [12]. Furthermore, IBS, a functional disorder, has overlapping symptoms with IBD, but lacks the inflammatory changes seen on endoscopy [13]. Lastly, colorectal cancer must be ruled out, given its potential to present with weight loss, anemia, and changes in bowel habits similar to IBD [14].

Differentiating IBD From IBS

Distinguishing between IBD and IBS is crucial due to their distinct pathophysiology, treatment, and prognosis. While both share abdominal symptoms, key differences exist [15]. IBD, encompassing CD and UC, features chronic gastrointestinal inflammation with potential complications such as strictures and fistulas [5]. In contrast, IBS lacks the inflammatory changes seen in IBD [16]. Diagnostic tests aid differentiation; IBD endoscopies show inflammation, ulcers, while IBS endoscopies appear normal [17]. IBD biopsies reveal inflammatory infiltrates, distinct from normal IBS biopsies [13]. Elevated inflammatory markers, CRP and ESR, are more common in IBD [18]. Extraintestinal manifestations such as arthritis point to IBD [19]. Management strategies vary; IBD often requires immunosuppression and surgery [20].

Diagnostic Resources

Diagnosing IBD involves a combination of clinical assessment and specialized diagnostic tools. The gold standard, colonoscopy, allows direct visualization and biopsy collection from the colon [21]. Flexible sigmoidoscopy evaluates the rectum and sigmoid colon, while capsule endoscopy offers insights into the small intestine [22]. Radiological imaging via CT scans and MRIs provides detailed abdominal images, revealing inflammation, strictures, or abscesses [23]. Barium studies visualize the small intestine's condition [24]. Blood tests identify inflammation markers such as CRP and ESR, while stool tests detect intestinal inflammation markers and rule out infections [25]. Microscopic examination of biopsy samples confirms chronic inflammation and other IBD-specific changes [26]. Specialized IBD clinics in tertiary care hospitals offer comprehensive diagnostic services, often utilizing a multidisciplinary approach [27].

Symptom Variations

Symptoms of IBD vary widely among patients, influenced by the disease type and inflammation location. Common symptoms include abdominal pain, diarrhea, and fatigue [28]. UC often leads to continuous symptoms such as bloody diarrhea, while CD can cause patchy inflammation, resulting in abdominal cramping and even extraintestinal symptoms such as joint pain [29]. External factors, such as stress or diet, can exacerbate symptoms, and complications such as strictures introduce new challenges like bowel obstruction [5]. Recognizing this variability is essential for accurate diagnosis and treatment.

Staging and Severity

In UC, severity ranges from mild symptoms with fewer than four daily stools to severe symptoms with over six bloody stools and systemic signs [30]. Moderate severity in UC is characterized by more than four stools daily with minimal systemic toxicity [31]. CD categorization is based on the disease's location and complications, such as inflammation in a single intestine segment or the presence of fistulas and abscesses [32]. Both UC and CD are also staged by the extent of involvement, which is crucial for guiding treatment and predicting prognosis [8].

Management

Medical Management

The medical management of IBD follows a stepwise medication approach, beginning with milder treatments and escalating to more potent therapies based on disease severity and patient response [33]. For moderate to severe cases, aggressive initial treatment is often recommended, involving high-efficacy medications or combination therapies to induce remission rapidly [34].

A range of medications is available for IBD management, including aminosalicylates, corticosteroids, immunomodulators, and biologic agents [35]. Selection depends on IBD type, symptom severity, and complications. Shared decision-making is crucial in IBD management, involving collaboration between healthcare providers and patients to discuss treatment options, risks, and alternatives [36]. This ensures well-informed and satisfactory treatment choices.

Regular monitoring and follow-up are essential, encompassing clinical assessments, lab tests, and endoscopic evaluations to maintain disease control and prevent complications [27].

Surgical Management

For patients with CD, surgical intervention becomes a consideration when medical treatments prove ineffective or complications arise. Common surgical procedures for CD include resection of affected bowel segments, such as ileocolic resection, which involves removing the diseased portion and reconnecting healthy segments [37]. While surgery can improve quality of life, it is not curative, and disease recurrence in other parts of the intestine is possible. Other surgical options for CD encompass strictureplasty to widen narrowed segments without removal, as well as procedures to address fistulas or abscesses [38].

In contrast, surgical approaches for UC can offer a potential cure. The most definitive surgery for UC is total proctocolectomy with ileal pouch-anal anastomosis (IPAA), removing the colon and rectum and creating a pouch from the small intestine's end connected to the anus for near-normal bowel function [39]. Another option is total proctocolectomy with ileostomy, where the small intestine's end is brought out to the abdomen's surface, creating a stoma for waste collection in an external pouch [40]. Surgical decisions depend on the patient's health, age, and preferences.

Collaboration and Guidelines

Effective IBD management requires collaboration among various healthcare professionals, ensuring comprehensive care [27]. Gastroenterological societies such as the American College of Gastroenterology (AGA), European Crohn’s and Colitis Organisation (ECCO), and British Society of Gastroenterology (BSG) have developed guidelines for IBD diagnosis, treatment, and long-term management [41]. These guidelines standardize care, promoting evidence-based practices. Additionally, patient advocacy groups work with medical professionals to develop patient-centered guidelines, emphasizing the patient's perspective. Adherence to these guidelines optimizes patient care and outcomes.

Upcoming Perspectives

The landscape of IBD management is rapidly evolving, presenting numerous promising avenues. Advances in genomics and molecular biology are driving personalized medicine, tailoring treatments based on individual genetic and molecular profiles [42]. Research on the gut microbiome suggests therapeutic potential from interventions such as probiotics, prebiotics, and fecal microbiota transplantation [43]. Emerging therapies, including novel biologics and small molecules, are targeting specific inflammatory pathways, providing precise treatment options with potentially fewer side effects [44]. The rise of digital health, encompassing wearable devices, telemedicine, and mobile apps, is revolutionizing patient monitoring and care, facilitating real-time feedback and remote consultations [45].

Multi-disciplinary Approach in Management of IBD

A multi-disciplinary approach to IBD management integrates specialists from various fields for comprehensive care. Gastroenterologists guide diagnosis and treatment [46]. Colorectal surgeons address surgical needs and complications [47]. Dietitians offer nutritional guidance, while psychologists address mental health concerns [48]. Radiologists and pathologists contribute to diagnosis and monitoring [49]. IBD nurses provide patient education and support, and pharmacists ensure optimal medication management [50].

Nutritional interventions

Exclusive Enteral Nutrition (EEN)

EEN is a nutritional therapy primarily utilized in managing CD, a subtype of IBD. It involves administering a liquid formula diet that provides essential nutrients while excluding regular foods [51]. This formula can be delivered orally or via a nasogastric tube.

EEN's exact mechanism of action is not fully understood, but it is believed to modulate the gut microbiome, reduce inflammation, and promote mucosal healing [52]. EEN has proven as effective as corticosteroids in inducing remission in pediatric CD patients, making it a preferred first-line therapy due to its lack of steroid-related side effects [53].

In adults, while EEN is effective, its acceptance is limited by factors such as formula taste and the social implications of not consuming regular food. Nevertheless, it remains vital, particularly for corticosteroid-resistant or corticosteroid-intolerant patients [54].

EEN's benefits extend beyond remission induction; it can improve nutritional status, promote growth in children, and enhance bone health. Despite these advantages, long-term EEN use is challenging due to issues with palatability and patient adherence.

Low Fermentable, Oligosaccharides, Disaccharides, Monosaccharides, and Polyols (FODMAP) Diet

Given the symptom overlap between IBS and IBD, a low FODMAP diet has been explored for IBD patients, especially those with IBS-like symptoms [55]. The low FODMAP diet, initially developed for IBS, restricts short-chain carbohydrates poorly absorbed in the small intestine [56]. While it can alleviate functional gastrointestinal symptoms, it does not treat the causes of IBD [57]. Implementing this diet requires guidance and continued follow-up from a trained nutritionist to ensure nutritional adequacy [58].

Anti-inflammatory Diets

Anti-inflammatory diets focus on foods with anti-inflammatory properties, such as fatty fish, berries, and green leafy vegetables, while limiting processed foods and sugars [59]. The Mediterranean diet, known for its anti-inflammatory benefits, has been linked to reduced inflammation markers [60]. The specific carbohydrate diet (SCD) restricts complex carbohydrates believed to exacerbate gut inflammation in IBD [61]. While promising, robust evidence for these diets in IBD is still emerging, and individualized guidance from a dietitian is crucial [62].

Specific Dietary Components

Exploring specific dietary components in the context of IBD, this review delves into the impact of dietary fiber, N-3 polyunsaturated fatty acids (PUFAs), and EEN. Dietary fiber, sourced from plants, supports gut health by promoting beneficial bacteria and producing anti-inflammatory short-chain fatty acids [63]. Recent studies indicate that, while IBD patients were traditionally advised to limit fiber during flare-ups, higher fiber intake during remission might mitigate future flare risks [64]. N-3 PUFAs, found in fatty fish, exhibit anti-inflammatory properties and compete with pro-inflammatory omega-6 fatty acids [65]. However, the efficacy of omega-3 supplements in IBD remains inconclusive. EEN utilizes a liquid formula as the sole nutrition source, offering therapeutic benefits by modulating the gut microbiome, reducing inflammation, and promoting mucosal healing. Despite challenges, EEN proves valuable, particularly when medications are unsuitable [66].

Mechanism of Action

This review delves into the complex connections between dietary components and IBD, focusing on the direct modification of the gut microbiome, metabolite production, and the immunological role of bile acid profiles [67]. Dietary interventions can reshape the gut microbiome's composition and function, where high-fiber diets foster beneficial bacteria growth, yielding anti-inflammatory short-chain fatty acids, while saturated fat and sugar-rich diets exacerbate harmful bacteria, worsening IBD symptoms. Probiotics introduce beneficial bacteria to restore microbial balance, with ongoing research into their effectiveness [68]. The gut microbiome's metabolism of dietary components yields metabolites such as butyrate, pivotal for gut health due to its anti-inflammatory properties and contribution to barrier integrity [69]. Bile acids, synthesized in the liver, function in fat digestion and immunological regulation. Secondary bile acids influence immune responses via specific receptors, impacting gut inflammation. In IBD, altered bile acid metabolism contributes to disease progression [70]. This insight into intricate mechanisms unveils novel therapeutic avenues in IBD management.

Comparative analysis

EEN

EEN is a therapeutic dietary approach involving a liquid formula as the sole source of nutrition for six to eight weeks [71]. Effective in pediatric CD remission, EEN's mechanism modulates the microbiome, reduces inflammation, and promotes healing. The absence of specific antigens might enhance its efficacy. Implementation challenges arise from the restrictive diet and taste aversion to the formula. Some patients may require nasogastric tube feeding for adequate intake [72]. Despite challenges, EEN is a vital non-pharmacological intervention in IBD management, especially when traditional medications falter [66].

Low FODMAP Diet

The low FODMAP diet targets fermentable carbohydrates poorly absorbed in the small intestine [56]. Such compounds can cause symptoms such as bloating, especially in individuals with sensitive guts or conditions such as IBS. For IBD patients, a low FODMAP diet has the potential to reduce symptoms and enhance the quality of life [57]. However, prolonged restriction might decrease beneficial gut bacteria, emphasizing the need for professional guidance [73].

Potential risks

EEN

EEN is a therapeutic intervention for IBD remission, notably in pediatric CD patients [71]. While effective, EEN comes with concerns. Nutritional deficiencies, weight loss, and growth retardation are risks due to limited liquid formula intake. Monotony can lead to taste fatigue, reduced appetite, and adherence challenges, especially in adolescents and adults [74]. The psychological impact of not eating regular food can cause social isolation, highlighting the need for careful monitoring and risk management [72].

Dietary Management of IBD

Dietary management is crucial for IBD care, aiming to alleviate symptoms and enhance well-being. While modifications offer benefits, they carry risks. Dietary changes without professional guidance can cause deficiencies, especially in restrictive diets [62]. Overly restrictive diets may limit food diversity, affecting gut health. Extreme diets risk malnutrition, weakened immunity, and disordered eating [75]. A balanced, individualized approach guided by professionals is vital to maximize dietary intervention benefits and minimize risks in IBD.

General Nutritional Advice for IBD

Essential in IBD care, general nutritional advice supports overall health [76]. While recommendations vary, key principles can reduce risks. Hydration prevents dehydration, vital during flares. Nutrient-rich foods provide essential vitamins and minerals, aiding immunity and healing. Fiber intake, limited during inflammation, demands caution [77]. Probiotics aid gut balance; alcohol moderation prevents gut barrier disruption [78]. Due to the individual nature of IBD, it is advisable to consult with a registered dietitian or a healthcare provider, such as a gastroenterologist or primary care provider, who has extensive expertise in assessing nutritional needs and providing customized advice tailored to each patient's requirements.

Challenges and Considerations

Managing IBD is complex due to varied presentations, treatment responses, and dietary tolerances. Genetics, the microbiome, and the environment create unique profiles. Personalized dietary counseling is essential [79]. Nutrition challenges arise from reduced intake, malabsorption, and restrictions. Malnutrition affects immunity and health. Inflammation increases nutrient needs, leading to deficiencies. Custom interventions and supplements are crucial [80]. Gastrointestinal tolerance is key. Inflammation disrupts function, altering sensitivity and motility. Diet changes can worsen symptoms, demanding careful choices [81]. IBD's impact extends beyond physical symptoms, causing anxiety, depression, and isolation. Youth are impacted by the condition, making educational interventions, counseling, and ongoing support essential [82]. The complexity of adhering to dietary guidelines is shaped by individual preferences and the nature of the disease itself. The cultural backdrop also plays a significant role in influencing dietary choices [75]. Despite these complexities, there is a lack of standardized dietary guidelines, and responses to treatment can vary depending on the subtype of the condition. Personalized approaches are vital [83]. Long-term data on interventions is limited. Understanding requires follow-up. Collaboration among researchers, healthcare, and patients is needed [84]. Patient preferences matter. Discussions consider culture, beliefs, and choices. Patient-centered care enhances engagement [85].

Interactions With Medications

A "customized diet plan" in managing IBD integrates dietary interventions and medication regimens [81]. Tailored recommendations, based on medical history, disease severity, and medication response, optimize outcomes. Collaborative discussions between providers yield personalized strategies that enhance treatment [86]. Dietary adjustments may address nutrient depletions from medications such as corticosteroids. Components' impact on absorption, metabolism, and side effects are considered [87]. Customization minimizes adverse interactions and maximizes dietary benefits in holistic IBD management.

Cultural Considerations

Considering "cultural food customs and beliefs" is vital in IBD dietary management. Cultural norms shape choices, necessitating sensitivity [88]. Preferences affect the adoption of changes. Inclusive plans that respect culture foster trust [89]. Understanding "acceptance and social dynamics" is pivotal [84]. Social interactions impact adherence. Coping strategies for social challenges are crucial. Acknowledging dynamics empowers patients and supports successful dietary management [90].

Cost Implications

The consideration of "cost implications" is paramount when addressing the dietary management of IBD. Implementing specific dietary interventions for IBD may entail financial considerations, including the costs associated with purchasing specialized foods, and supplements, and consulting with registered dietitians or healthcare professionals [88]. The affordability of dietary modifications can significantly impact patients' ability to adhere to recommended dietary changes. Patients may encounter challenges in balancing the costs of dietary adjustments with their financial resources and other medical expenses. Healthcare providers should engage in open discussions with patients about potential cost-related barriers, explore cost-effective alternatives, and provide guidance on making informed decisions while prioritizing nutritional needs [89]. By addressing cost implications, healthcare professionals contribute to the development of sustainable and accessible dietary management strategies that align with patients' financial capacities and overall well-being.

Recommendations for Future Research

Exploring various dimensions in the context of IBD management through dietary interventions can offer valuable insights to enhance patient care. Longitudinal studies analyzing dietary patterns over extended periods can shed light on the long-term effects of dietary strategies on disease progression, symptom management, and health outcomes [88]. Such studies can identify patterns linked to reduced disease activity and improved quality of life. Understanding the interaction between nutrients and gut microbiota composition is another avenue that holds promise for refining dietary interventions [90]. Investigating how specific nutrients influence the gut microbiota can provide insights into novel therapeutic targets and dietary approaches to modulate inflammation. Moreover, exploring the impact of microbial metabolites from dietary components on intestinal health and immune responses can provide a comprehensive view of diet-microbiome interactions [84]. By uncovering these relationships, personalized dietary recommendations can be tailored to individuals' microbiome profiles and nutritional needs. Additionally, recognizing cultural norms and integrating them into dietary recommendations can enhance the acceptability and feasibility of dietary changes [91]. Investigating culturally tailored interventions that align with individuals' culinary traditions can empower patients to adopt sustainable dietary modifications. This approach can address not only disease outcomes but also the social and cultural dimensions of patients' lives [92]. By embracing these multifaceted strategies, researchers can contribute to a more holistic and patient-centered approach to IBD care.

## Conclusions

This review delves into the connection between diet and IBD. It examines how dietary changes can help manage IBD, highlighting various nutritional strategies, potential risks, and future possibilities. This makes it an essential tool for doctors, researchers, and patients. The data suggest that it is crucial to tailor diet plans to individual IBD patients, considering their specific disease types and cultural preferences. Moreover, the review discusses different dietary components, from specialized nutrition like EEN to the effects of omega-3 fatty acids, providing practical advice and areas for further research.

Blending the latest research with practical medical advice, this review helps healthcare professionals make informed decisions about IBD dietary management, including dietitians in a management team. As more people are diagnosed with IBD, the insights offered here are invaluable for medical professionals aiming to understand and manage the disease's dietary aspects better. Encouraging patients to participate actively in their care, the review underscores the vital role diet plays in IBD management. Overall, it points toward a comprehensive, patient-centered approach to IBD care, emphasizing the importance of dietary evaluation and adherence in treatment and management strategies.
